# Medical Items

**Published:** 1894-12

**Authors:** 


### *MEDICAL ITEMS.*

---

Dr. George L. Alexander and Miss Dorothy B. Wright, of Forsyth, Ga., were married November 21st. Congratulations.

---

Dr. Thos. M. McIntosh, of Thomasville, has been appointed Principal Physician of the Penitentiary, to succeed Dr. O'Daniel.

---

A late act of the New York legislature established the Craig Colony for Epileptics at Mount Morris, near Buffalo. Our New York correspondent, Dr. W. P. Spratling, has been elected Superintendent. We will hear from Dr. Spratling in the future about the workings of this interesting institution.

---

The last (November) issue of the Alabama *Medical and Surgical Age* contains an interesting article by Dr. Thos. A. Means, of Montgomery, entitled "Reminiscences of Dr. J. Marion Sims—Incidents of His Early Professional Life." The incidents here related were told by Dr. Sims to Dr. Means about two years before the death of the former. They are the same as have been described by Dr. Sims in his "Story of my Life." Any one who has not read this entertaining book will be interested by this article in the *Age*. He may be encouraged also and taught the valuable lesson of patience and perseverance.

---

In the death of Dr. William Goodell, of Philadelphia, October 27th, the American profession of gynecology lost one of its brightest representatives. Dr. Goodell was the father of that class of modern gynecologists who believe that a woman has something more than a uterus and its appendages. He was the greatest American exponent and advocate of conservative methods of treatment. Among his best papers was his "Nerve-Counterfeits of Uterine Disease," which called attention to the oft-made mistake of regarding the uterus and ovaries as the causes of all disease among women. In an able paper published about one year ago, he emphasized this common mistake in a paper entitled "The Great Medical Error of the Day." But he was not too conservative to recognize proper cases for radical methods, and in the discrimination he possessed a safe and reliable judgment which was unexcelled.

Dr. Goodell was born on the island of Malta. He was educated in Philadelphia, and nearly his entire professional life was spent there. In 1870 he became Lecturer on Obstetrics and Gynecology in the University of Pennsylvania, and a few years later was made Clinical Professor. The latter position he retained until one year ago, when failing health compelled him to resign. Dr. Goodell's chief medical work was his "Lectures on Gynecology."

---

TOO PROMISCUOUS.—The *Railway Age*, in its issue of November 16th, says:

The two thousand or so railway surgeons who belong to the National Association of Railway Surgeons may like to know why a score or so of their fellow-members met in Chicago last week to organize "The American Academy of Railway Surgeons." Here is the answer from an official source:

> The direct reason for the forming of the new organization is that the National Association of Railway Surgeons has become too promiscuous, and it was desired to have a more select body. The membership is to be limited to something less than two hundred, and will be confined to only the more eminent surgeons in railway service.

This ought to be satisfactory to the railway surgeons of the country. A few of their number have decided that the rest are "too promiscuous," and therefore proceed to constitute themselves "a more select body," with which the great majority of the profession is unworthy to associate. "We, the more eminent surgeons in railway service," is impressive, but it may not be convincing.—*The Railway Surgeon.*

